# Prediction of neoadjuvant chemotherapy efficacy and prognostic biomarker analysis in patients with triple-negative breast cancer

**DOI:** 10.3389/fphar.2025.1553831

**Published:** 2025-03-12

**Authors:** Xiao-Wen Liao, Jia-Bin Gao, Hong Sun, Hong-Dan Chen, Min-Hui Zheng, Lei Han, Xiao-Geng Chen, Yu-Nan Su, Ding-Long Pan, Min Wu, Shuang-Long Cai, Xiuquan Lin, Guo-Zhong Chen

**Affiliations:** ^1^ Department of Radiation Oncology, The Second Affiliated Hospital of Fujian Medical University, Quanzhou, Fujian, China; ^2^ General Surgery Department of Pengyang County People’s Hospital, Guyuan, Ningxia, China; ^3^ Department of Pharmacy, Fujian Provincial Hospital, Shengli Clinical Medical College of Fujian Medical University, Fuzhou University Affiliated Provincial Hospital, Fuzhou, Fujian, China; ^4^ First Department of Cadre Clinic, Fujian Provincial Hospital, Shengli Clinical Medical College of Fujian Medical University, Fuzhou University Affiliated Provincial Hospital, Fuzhou, Fujian, China; ^5^ Oral and Maxillofacial Surgery Thyroid and Hernia Surgery, Fujian Provincial Hospital, Shengli Clinical Medical College of Fujian Medical University, Fuzhou University Affiliated Provincial Hospital, Fuzhou, Fujian, China; ^6^ Department of Breast Surgery, Fujian Provincial Hospital, Shengli Clinical Medical College of Fujian Medical University, Fuzhou University Affiliated Provincial Hospital, Fuzhou, Fujian, China; ^7^ Department of Emergency, The Second Affiliated Hospital of Fujian Medical University, Quanzhou, Fujian, China; ^8^ Department for Chronic and Noncommunicable Disease Control and Prevention, Fujian Provincial Center for Disease Control and Prevention, Fuzhou, Fujian, China

**Keywords:** triple-negative breast cancer, neoadjuvant chemotherapy, efficacy prediction, prognostic analysis, nomogram

## Abstract

**Background:**

Neoadjuvant chemotherapy has become a common and effective treatment modality for triple-negative breast cancer (TNBC). The primary goal is to reduce the size of the primary tumor, enabling breast-conserving surgery, axillary preservation, and a transition to operability, thereby providing patients with more therapeutic options. Although neoadjuvant chemotherapy (NAC) has demonstrated favorable outcomes in clinical practice, predicting its efficacy and prognostic value in TNBC remains a key challenge in current clinical research.

**Methods:**

This study included 248 TNBC patients who received NAC at two breast cancer treatment centers. By employing a modeling validation approach, we aim to explore predictors of treatment efficacy and potential prognostic biomarkers associated with NAC.

**Results:**

In the multivariable analysis of the training set, the factors predicting the pathological complete response (pCR) to NAC in TNBC patients include high biopsy-sTILs expression, biopsy-Ki67 > 20%, and positive expression of biopsy-androgen receptor (AR). The factors predicting disease-free survival (DFS) are ypN3, high postoperative sTIL expression, receipt of postoperative radiotherapy, and effective NAC. The factors predicting overall survival (OS) include ypN2, ypN3, high postoperative sTIL expression, postoperative Ki67 > 20%, receipt of postoperative radiotherapy, and effective NAC. The C-indices in the training and validation sets for the prediction of pCR using the nomogram were 0.729 and 0.816, respectively. The C-indices for predicting DFS were 0.895 and 0.865, respectively. The C-indices for predicting OS were 0.899 and 0.860, respectively.

**Conclusion:**

This study established and validated a nomogram model predicting the pCR, DFS, and OS in TNBC patients undergoing NAC. This model demonstrates good discrimination and accuracy.

## 1 Introduction

As the incidence of breast cancer continues to rise globally, it has become one of the leading causes of cancer-related deaths among women worldwide (Bray et al., 2024). Triple-negative breast cancer (TNBC) is a subtype characterized by the lack of expression of estrogen receptors (ER), progesterone receptors (PR), and human epidermal growth factor receptor 2(HER2). Due to the absence of clear molecular therapeutic targets, chemotherapy remains the primary treatment modality for TNBC (Siegel et al., 2023). Given that TNBC is an extremely aggressive and heterogeneous type of breast cancer, accounting for approximately 10%–20% of invasive breast cancers (Cortes et al., 2023), treatment options are often limited, and the recurrence rate is high, resulting in poorer prognoses compared to other subtypes of breast cancer (Kumar et al., 2023). In recent years (Gradishar et al., 2024; Loibl et al., 2024), neoadjuvant chemotherapy (NAC) has become a common treatment approach for TNBC. This therapy aims to downstage the cancer, transforming “inoperable breast cancer” into “operable breast cancer,” and converting surgeries that previously required mastectomy into breast-conserving procedures. Furthermore, neoadjuvant treatment can provide early information on the tumor’s sensitivity to drugs, offering patients and clinicians more clinical strategies for subsequent treatment.

Currently, there are no widely approved biomarkers for predicting NAC efficacy and patients’ prognosis. Therefore, identifying biomarkers that can distinguish between good and poor responses at baseline would be crucial for treatment decisions in TNBC patients. Pathological complete response (pCR) is a commonly used effective method for evaluating the efficacy of NAC. Meta-analyses have shown that (Conforti et al., 2021) achieving pCR significantly improves survival outcomes for breast cancer patients, with those reaching pCR exhibiting markedly prolonged event-free survival (EFS) and overall survival (OS) compared to those who do not achieve pCR. Thus, achieving pCR during surgery is often considered a surrogate marker for long-term survival (Cortazar et al., 2014). However, some studies indicate that increasing pCR rates does not necessarily correlate with improved prognosis (Shepherd et al., 2022), as a small proportion of patients who achieve pCR may still experience distant metastasis (Pusztai et al., 2024). Consequently, predicting the efficacy and prognosis of NAC requires a more comprehensive analysis.

This study aims to establish a nomogram model to analyze potential predictive and prognostic biomarkers affecting the rates of pCR, disease-free survival, and overall survival in real-world TNBC patients undergoing neoadjuvant chemotherapy. The findings will provide valuable insights for better clinical diagnosis and treatment of TNBC patients receiving neoadjuvant chemotherapy.

## 2 Materials and methods

### 2.1 Patients and data collection

A total of 248 cases of primary unilateral invasive breast cancer diagnosed between 1 January 2015, and 31 December 2020, were collected from the Second Affiliated Hospital of Fujian Medical University and Fujian Provincial Hospital. All cases were female.

Inclusion criteria: 1) Histopathological diagnosis of invasive ductal carcinoma with immunohistochemistry showing negative results for ER, PR, and HER2, classifying them as triple-negative breast cancer. 2) Initial treatment with neoadjuvant chemotherapy. 3) Surgical intervention following neoadjuvant chemotherapy, followed by adjuvant therapy.

Exclusion criteria: 1) Patients diagnosed with stage IV breast cancer at initial presentation. 2) Patients without severe cardiac, hepatic, or renal dysfunction prior to chemotherapy and without contraindications to chemotherapy. 3) Incomplete or missing clinical, pathological, treatment, or follow-up data.

Clinical data collected included age, menstrual status, family history, surgical methods, chemotherapeutic agents used during neoadjuvant and postoperative adjuvant therapy, receipt of postoperative radiotherapy, efficacy of neoadjuvant treatment, and recurrence/metastasis status. The selection of all NAC patients, radiotherapy patients, radiotherapy target areas, and radiation doses were based on the NCCN guidelines for breast cancer. Pathological data included the clinical T staging and axillary lymph node N staging before neoadjuvant treatment, histological grading, stromal tumor-infiltrating lymphocytes (sTIL), expression levels of ER, PR, HER2, Ki-67, and androgen receptors (AR) from pre-treatment biopsies and post-surgical specimens; as well as the presence of lymph-vascular invasion, CK5/6, and EGFR status after surgery.

### 2.2 Diagnosis and immunohistochemistry technique

Immunohistochemical (IHC) evaluations of ER, PR, and HER2 were conducted according to the guidelines provided by the American Society of Clinical Oncology (ASCO) and the College of American Pathologists (CAP). ER or PR was considered negative if less than 1% or 0% of tumor cell nuclei exhibited immunoreactivity (Allison et al., 2020). HER2 negativity was defined as IHC 0, IHC 1+, or IHC 2+ with negative fluorescence *in situ* hybridization (FISH) results (Wolff et al., 2018). AR, cytokeratin 5/6 (CK5/6), and epidermal growth factor receptor (EGFR) expression levels below 1% were classified as negative, while levels equal to or greater than 1% were classified as positive. The assessment of sTILs was performed based on internationally recognized standards. sTILs were categorized as low (≤10%), moderate (10% to ≤40%), or high (>40%). All immunohistochemical readings were validated by two blinded, trained pathologists.

### 2.3 Efficacy evaluation

The efficacy of neoadjuvant chemotherapy for breast cancer was evaluated according to the Response Evaluation Criteria in Solid Tumors (RECIST 1.1). Complete Response (CR): All target and non-target lesions disappeared, with all lymph nodes measuring <10 mm in diameter. Partial Response (PR): The sum of the diameters of target lesions decreased by at least 30% compared to baseline. Progressive Disease (PD): The sum of the longest diameters of target lesions increased by more than 20%, or an absolute increase of more than 5 mm, with the presence of new malignant lesions classified as PD. Stable Disease (SD): Target lesions that did not meet criteria for PR (decrease) or PD (increase). CR and PR are considered indicative of a response to treatment, while SD and PD are regarded as non-responders. Pathological Complete Response (pCR) is defined as ypT0N0/ypTisN0M0. In evaluating the efficacy of NAC, it is essential to consider both the primary lesion and lymph nodes. A primary breast lesion without invasive carcinoma and negative regional lymph nodes is defined as pCR.Disease-free survival (DFS) is defined as the time from diagnosis to local or regional recurrence, distant metastasis, death (including non-cancer-related deaths), or the last follow-up date (1 June 2024). Overall survival (OS) is defined as the time from diagnosis until death from any cause or the last follow-up date (1 June 2024). All patients were monitored primarily through telephone follow-ups or outpatient visits.

### 2.4 Follow-up

Patients diagnosed with breast cancer undergo a series of initial assessments after their first admission, including cranial and chest CT scans, breast ultrasound, abdominal ultrasound, and whole-body bone emission computed tomography(ECT) to rule out the possibility of distant metastasis. For the first three years post-surgery, follow-up is conducted every three months, involving chest X-rays or chest CT scans, breast ultrasounds, liver ultrasounds, and related tumor marker tests. From three to five years post-surgery, follow-ups occur every six months, maintaining the same procedures. For patients beyond five years, these checks will occur annually. If, during regular follow-up examinations, there are suspicions of possible bone or brain metastases, additional imaging such as whole-body bone ECT and cranial CT or MRI will be performed.

### 2.5 Statistical analysis

The statistical software used was SPSS version 22.0 and R version 4.0.0. Categorical data were expressed as n (%), and group comparisons were conducted using the χ^2^ test. A binary logistic regression model was employed to analyze the influencing factors of pathological complete response (PCR). Univariate and multivariate Cox regression analyses were performed to identify the factors affecting disease-free survival (DFS) and overall survival (OS). The “rms” package in R was used to construct a nomogram and receiver operating characteristic (ROC) curves for predictive performance analysis. Calibration curves were utilized to evaluate the model’s goodness of fit, with a significance level set at P < 0.05 indicating statistical significance.

## 3 Results

### 3.1 Characteristics of the study population

Among the 248 patients with triple-negative breast cancer (TNBC), 77 achieved a pathological complete response (PCR). The training cohort included 174 TNBC women, with a median follow-up time of 59.80 months. During the follow-up period in the training cohort, there were 53 disease-free survival (DFS) events and 51 deaths. The validation cohort comprised 74 TNBC women, with a median follow-up time of 65.59 months. In the validation cohort, there were 20 DFS events and 19 deaths. The demographic and clinical-pathological characteristics of patients in both the training and validation cohorts are presented in Table 1, indicating that the two groups are comparable.

**TABLE 1:** The demographic and clinical-pathological characteristics of patients in both the training and validation sets.

	Training sets	Validation sets	X^2^	P
	(n = 174)	(n = 74)		
Age at diagnosis			0.913	0.339
≤35 years	33 (18.97)	18 (24.32)		
>35 years	141 (81.03)	56 (75.68)		
Menstrual status			0.034	0.854
Premenopausal	106 (60.92)	46 (62.16)		
Postmenopausal	68 (39.08)	28 (37.84)		
Family history			0	0.984
No	153 (87.93)	65 (87.84)		
Yes	21 (12.07)	9 (12.16)		
Surgical approach			0.119	0.73
Radical surgery	151 (86.78)	63 (85.14)		
Breast-conserving surgery	23 (13.22)	11 (14.86)		
ypT staging			5.829	0.212
ypT0/Tis	58 (33.33)	33 (44.59)		
ypT1	56 (32.18)	20 (27.03)		
ypT2	37 (21.26)	11 (14.86)		
ypT3	21 (12.07)	7 (9.46)		
ypT4	2 (1.15)	3 (4.05)		
cT staging			2.876	0.09
cT1+cT2	64 (36.78)	19 (25.68)		
cT3+cT4	110 (63.22)	55 (74.32)		
ypN staging			5.189	0.158
ypN0	103 (59.2)	34 (45.95)		
ypN1	40 (22.99)	25 (33.78)		
ypN2	18 (10.34)	11 (14.86)		
ypN3	13 (7.47)	4 (5.41)		
cN staging			3.739	0.053
cN0	51 (29.31)	13 (17.57)		
cN+	123 (70.69)	61 (82.43)		
Post-histological grading			0.000	0.991
G1+G2	113 (64.94)	48 (64.86)		
G3	61 (35.06)	26 (35.14)		
Post-lymph-vascular invasion			0.003	0.955
No	124 (71.26)	53 (71.62)		
Yes	50 (28.74)	21 (28.38)		
Post-sTIL levels			0.382	0.826
Low	46 (26.44)	22 (29.73)		
Intermediate	40 (22.99)	15 (20.27)		
High	88 (50.57)	37 (50)		
Biopsy-sTIL levels			0.645	0.724
Low	59 (33.91)	28 (37.84)		
Intermediate	58 (33.33)	21 (28.38)		
High	57 (32.76)	25 (33.78)		
Post-Her2 levels			0.592	0.744
0	35 (20.11)	15 (20.27)		
1+	95 (54.6)	37 (50)		
2+	44 (25.29)	22 (29.73)		
Biopsy-Her2 levels			1.022	0.6
0	69 (39.66)	25 (33.78)		
1+	79 (45.4)	35 (47.3)		
2+	26 (14.94)	14 (18.92)		
Post-Ki67 levels			2.097	0.148
Ki67 ≤ 20	40 (22.99)	11 (14.86)		
Ki67 > 20	134 (77.01)	63 (85.14)		
Biopsy-Ki67 levels			0.621	0.43
Ki67 ≤ 20	68 (39.08)	25 (33.78)		
Ki67 > 20	106 (60.92)	49 (66.22)		
Post-CK5/6 levels			1.646	0.199
CK5/6 < 1	88 (50.57)	44 (59.46)		
CK5/6 ≥ 1	86 (49.43)	30 (40.54)		
Post-EGFR levels			2.096	0.148
EGFR<1	86 (49.43)	44 (59.46)		
EGFR≥1	88 (50.57)	30 (40.54)		
Post-AR levels			2.587	0.108
<1	101 (58.05)	51 (68.92)		
≥1	73 (41.95)	23 (31.08)		
Biopsy-AR levels			2.02	0.155
<1	121 (69.54)	58 (78.38)		
≥1	53 (30.46)	16 (21.62)		
Neoadjuvant chemotherapy drugs used			2.444	0.485
Anthracyclines	1 (0.57)	0 (0)		
Taxanes	1 (0.57)	0 (0)		
Anthracyclines + taxanes	140 (80.46)	56 (75.68)		
Combined with platinum	32 (18.39)	18 (24.32)		
Neoadjuvant + postoperative adjuvant chemotherapy drugs used			0.547	0.459
Anthracyclines + taxanes	139 (79.89)	56 (75.68)		
Combined with platinum	35 (20.11)	18 (24.32)		
Radiation therapy status			0.321	0.571
No	30 (17.24)	15 (20.27)		
Yes	144 (82.76)	59 (79.73)		
NAC response			1.341	0.247
Non-response groups	70 (40.23)	24 (32.43)		
Response groups	104 (59.77)	50 (67.57)		
NAC response			3.265	0.071
Non-pCR	126 (72.41)	45 (60.81)		
PCR	48 (27.59)	29 (39.19)		

### 3.2 Nomogram prediction model for PCR

**FIGURE 1:**
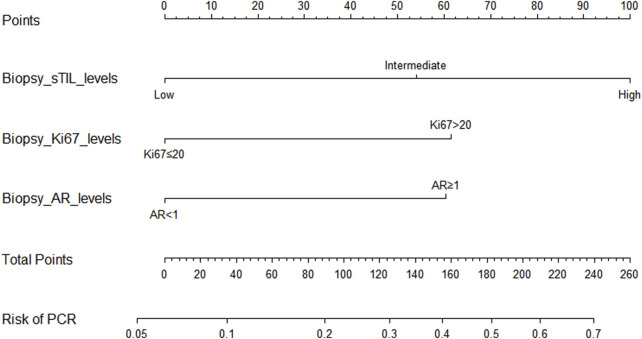
Nomogram for predicting the pathological complete response rate in triple-negative breast cancer patients receiving neoadjuvant chemotherapy.

**FIGURE 2:**
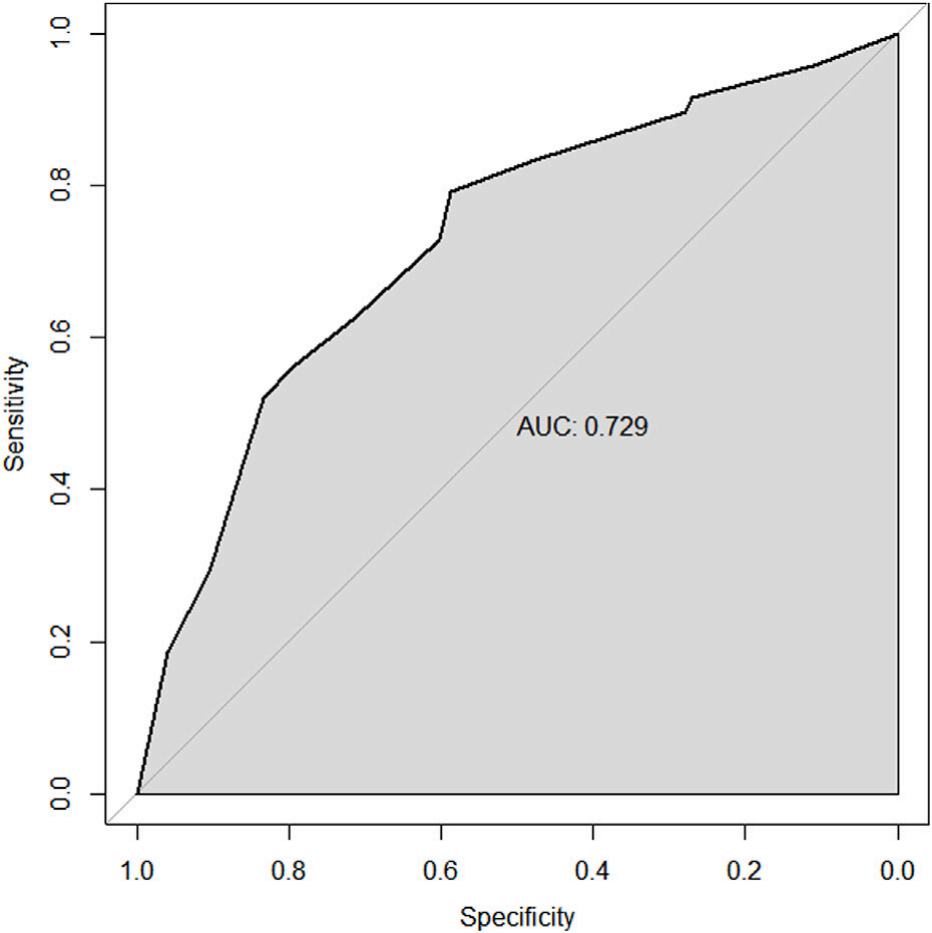
ROC curve of the training set.

**FIGURE 3:**
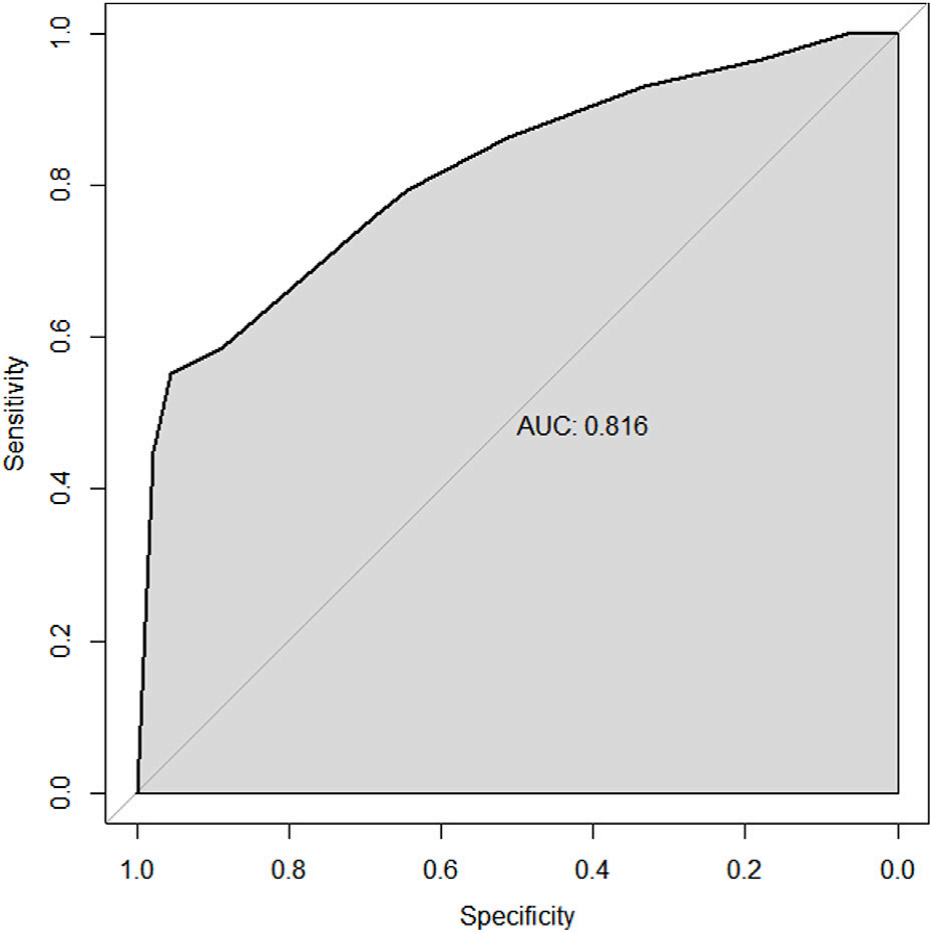
ROC curve of the validation set.

**FIGURE 4:**
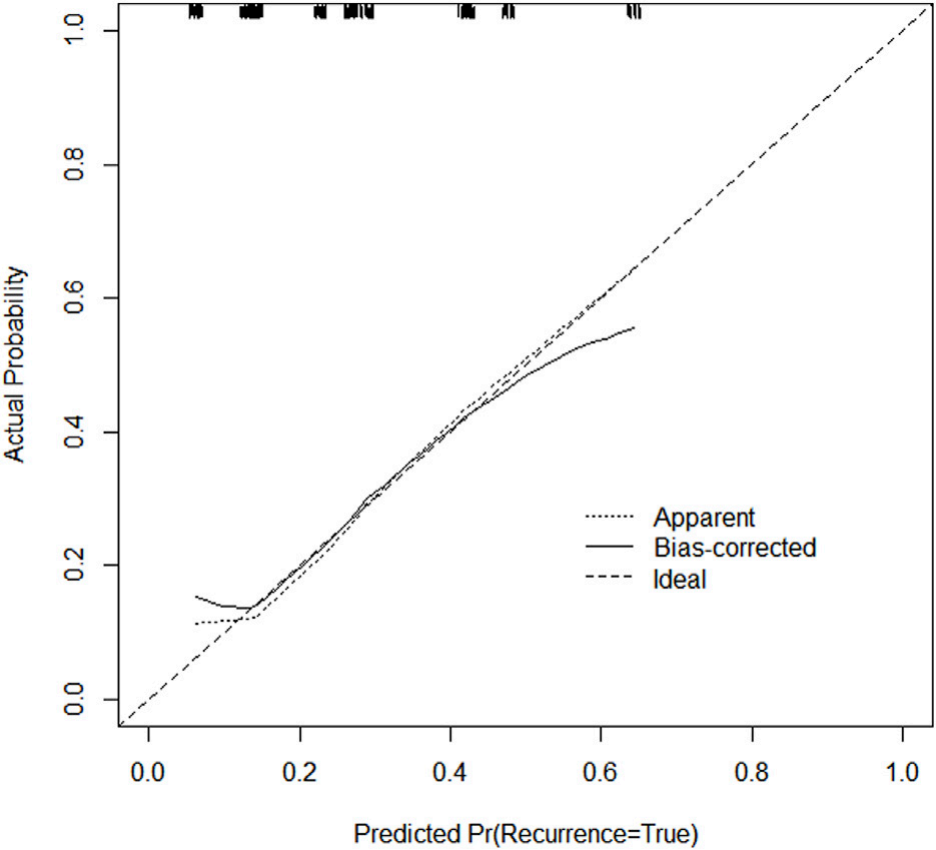
Calibration curve of the training set.

**FIGURE 5:**
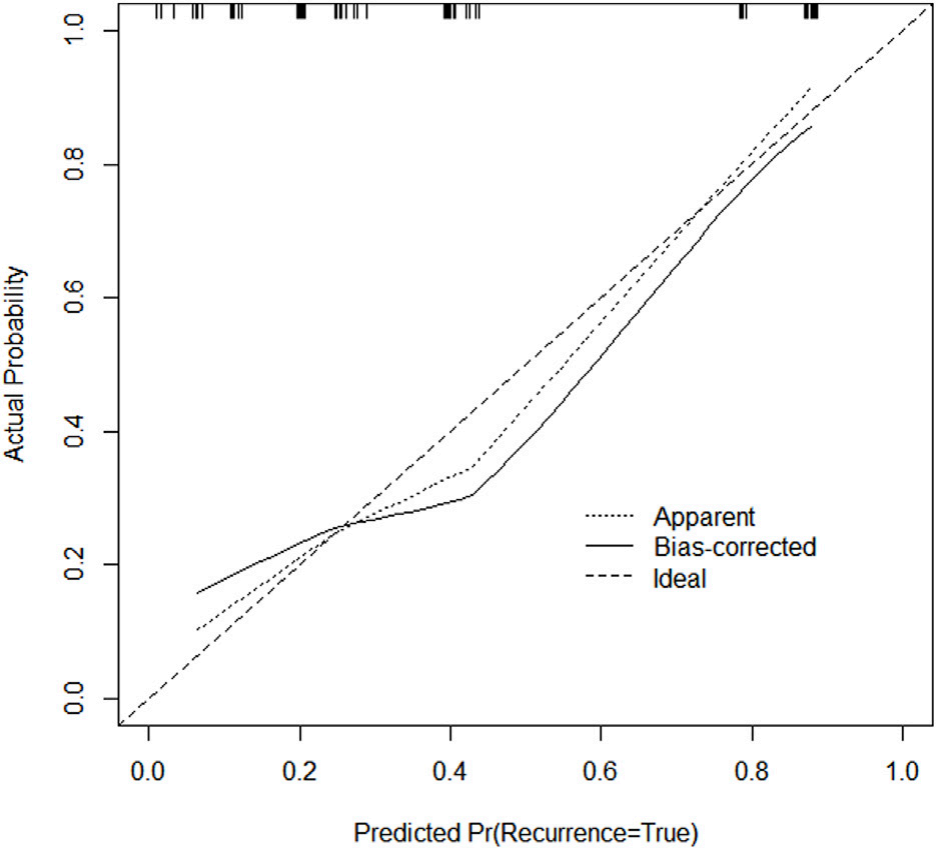
Calibration curve of the validation set.

Through univariate and multivariate variable analyses in the training set, the final variables predicting PCR in TNBC patients receiving neoadjuvant chemotherapy were high biopsy-sTILs expression, biopsy-Ki67 > 20%, and positive expression of biopsy-androgen receptor (AR) (see Table 2). Subsequently, a nomogram was developed incorporating these three variables (Figure 1), which we named the nomogram for predicting the pathological complete response rate in triple-negative breast cancer patients receiving neoadjuvant chemotherapy. The area under the curve (AUC) of the nomogram in both the training and validation groups reached an ideal consistency, with C-indices of 0.729 and 0.816 respectively (Figures 2, 3), and their calibration curves are shown in Figures 4, 5.

**TABLE 2:** Univariate and Multivariate Analysis of neoadjuvant efficacy in the training set TNBC patients.

Variables	PCR	Non-PCR	Univariate analysis		Multivariate analysis	
			X^2^	P	Or (95% CI)	P
Age at diagnosis			0.002	0.964		
≤35 years	24 (19.05)	9 (18.75)				
>35 years	102 (80.95)	39 (81.25)				
Menstrual status			0.07	0.792		
Premenopausal	76 (60.32)	30 (62.5)				
Postmenopausal	50 (39.68)	18 (37.5)				
Family history			0.872	0.351		
No	109 (86.51)	44 (91.67)				
Yes	17 (13.49)	4 (8.33)				
cT staging			3.957	0.047		
cT1+cT2	52 (41.27)	12 (25)			ref	
cT3+cT4	74 (58.73)	36 (75)			1.685 (0.749–3.789)	0.207
cN staging			2.299	0.129		
cN0	41 (32.54)	10 (20.83)				
cN+	85 (67.46)	38 (79.17)				
Biopsy-sTIL levels			10.593	0.005		
Low	50 (39.68)	9 (18.75)			ref	
Intermediate	43 (34.13)	15 (31.25)			2.017 (0.741–5.491)	0.17
High	33 (26.19)	24 (50.00)			3.507 (1.345–9.145)	0.01
Biopsy-Her2 levels			7.941	0.019		
0	58 (46.03)	11 (22.92)			ref	
1+	52 (41.27)	27 (56.25)			1.842 (0.775–4.382)	0.167
2+	16 (12.7)	10 (20.83)			2.114 (0.697–6.416)	0.186
Biopsy-Ki67 levels			5.52	0.019		
Ki67 ≤ 20	56 (44.44)	12 (25.00)			ref	
Ki67 > 20	70 (55.56)	36 (75.00)			2.411 (1.081–5.376)	0.031
Biopsy-AR levels			9.537	0.002		
<1	96 (76.19)	25 (52.08)			ref	
≥1	30 (23.81)	23 (47.92)			2.329 (1.099–4.936)	0.027
Neoadjuvant chemotherapy drugs used			1.468	0.69		
Anthracyclines	1 (0.79)	0 (0)				
Taxanes	1 (0.79)	0 (0)				
Anthracyclines + taxanes	100 (79.37)	40 (83.33)				
Combined with platinum	24 (19.05)	8 (16.67)				

### 3.3 Nomogram prognostic model for disease-free survival (DFS)

**FIGURE 6:**
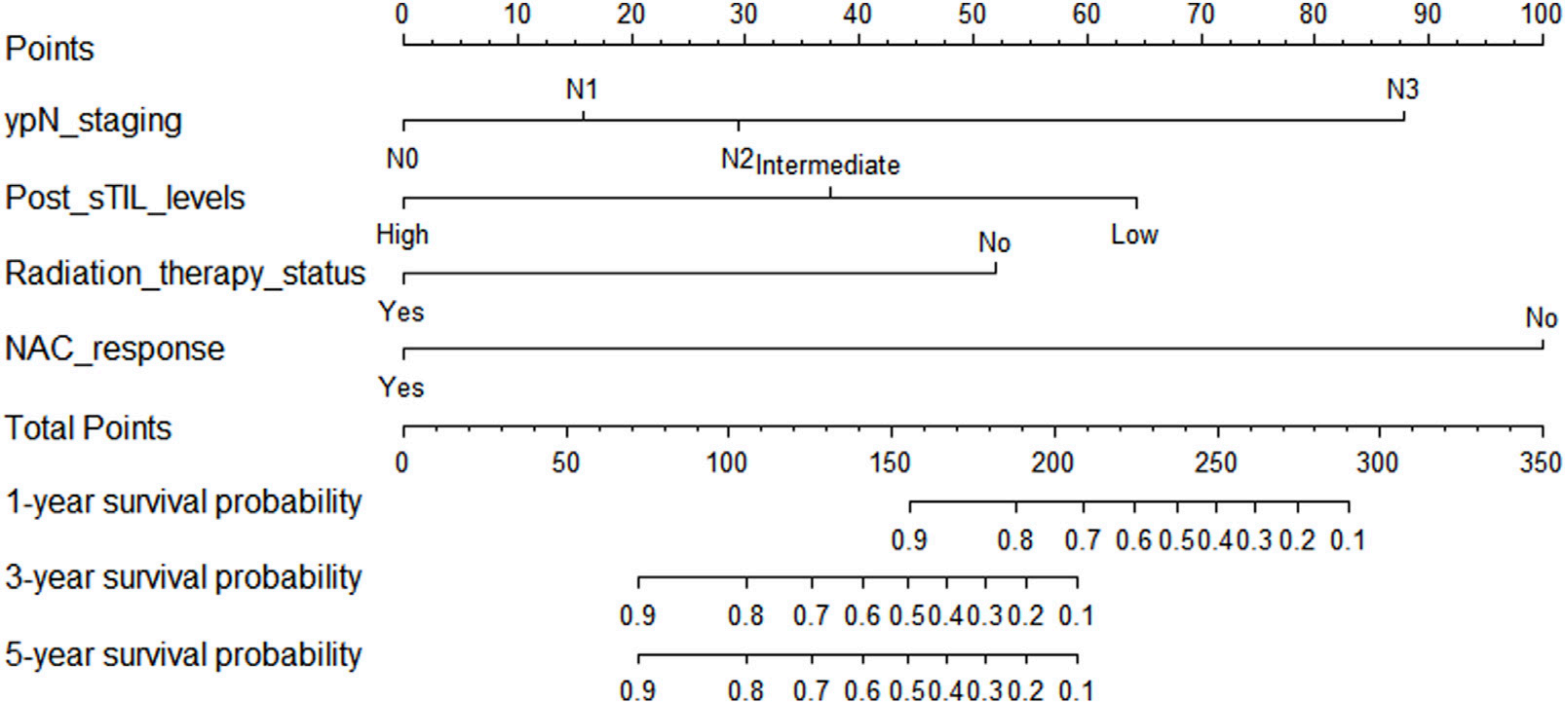
Nomogram for predicting disease-free survival in triple-negative breast cancer patients undergoing neoadjuvant chemotherapy.

**FIGURE 7:**
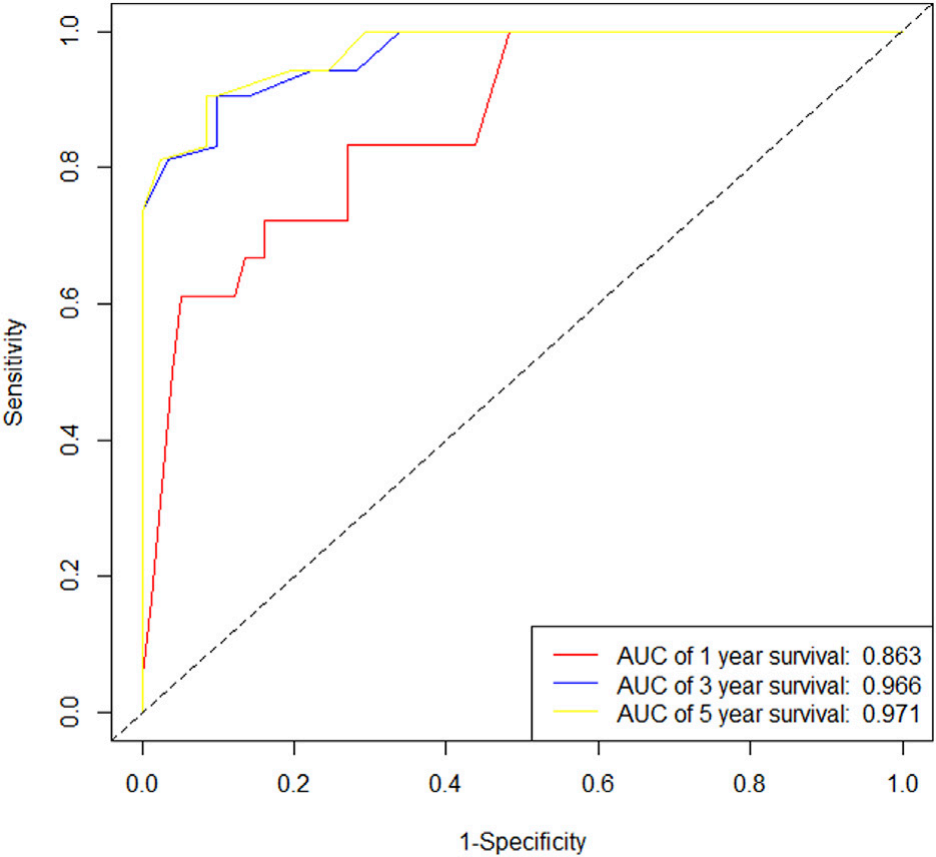
ROC curve of the training set.

**FIGURE 8:**
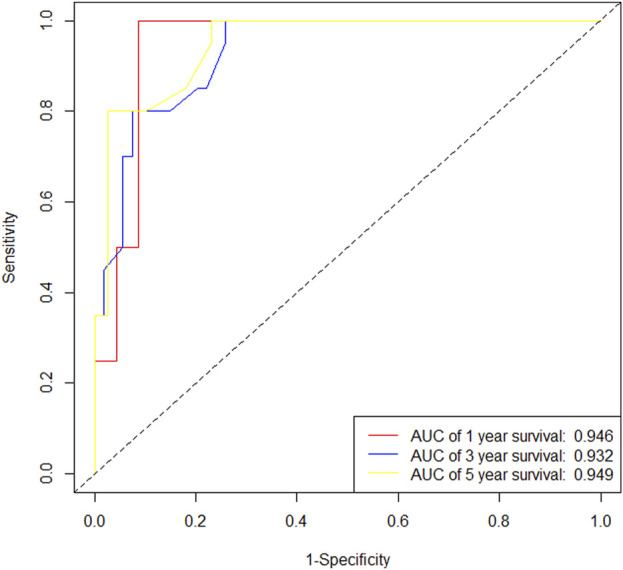
ROC curve of the validation set.

**FIGURE 9:**
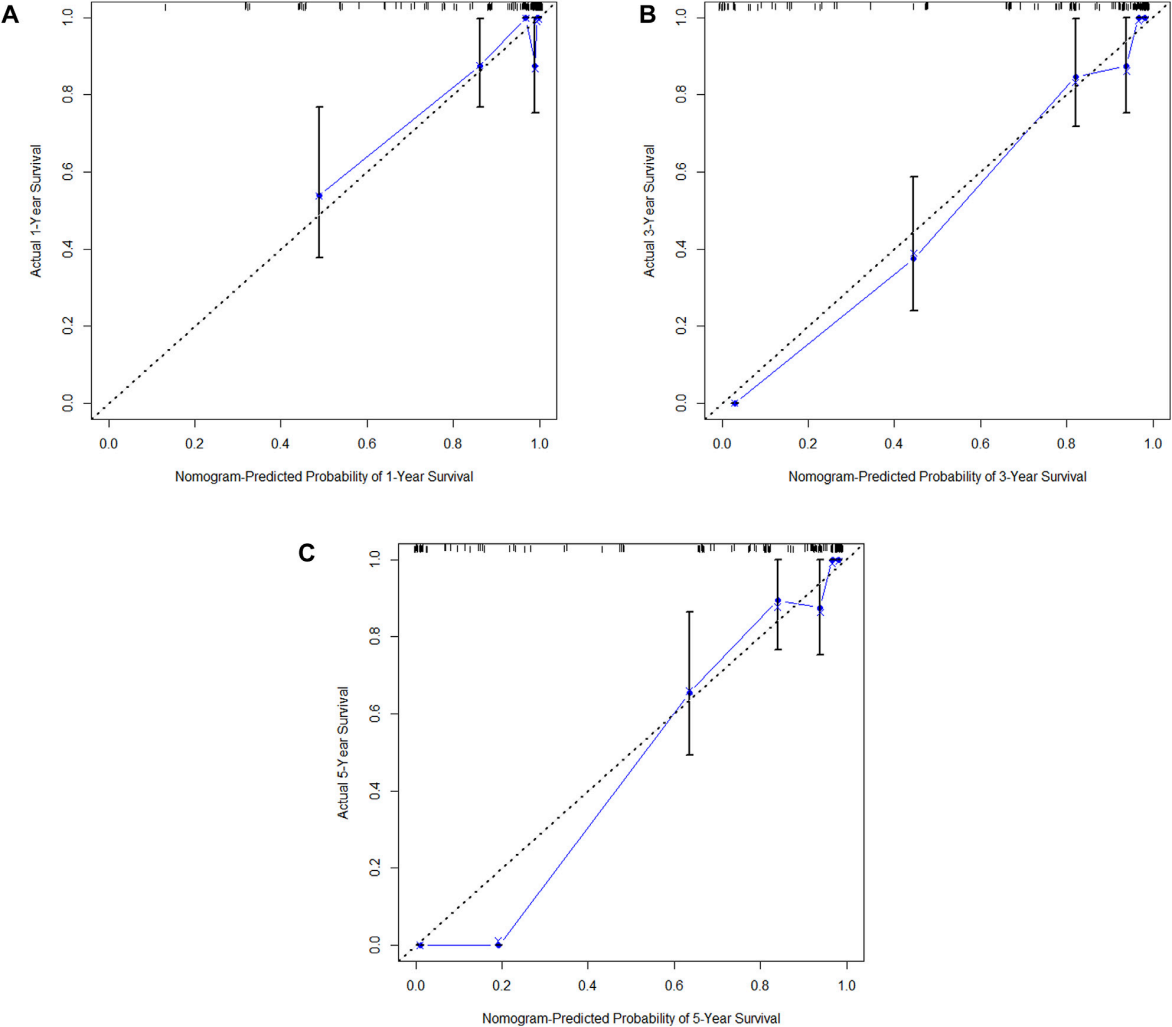
**(A)** Calibration curve of the training set at 1 year. **(B)** Calibration curve of the training set at 3 year. **(C)** Calibration curve of the training set at 5 year.

**FIGURE 10:**
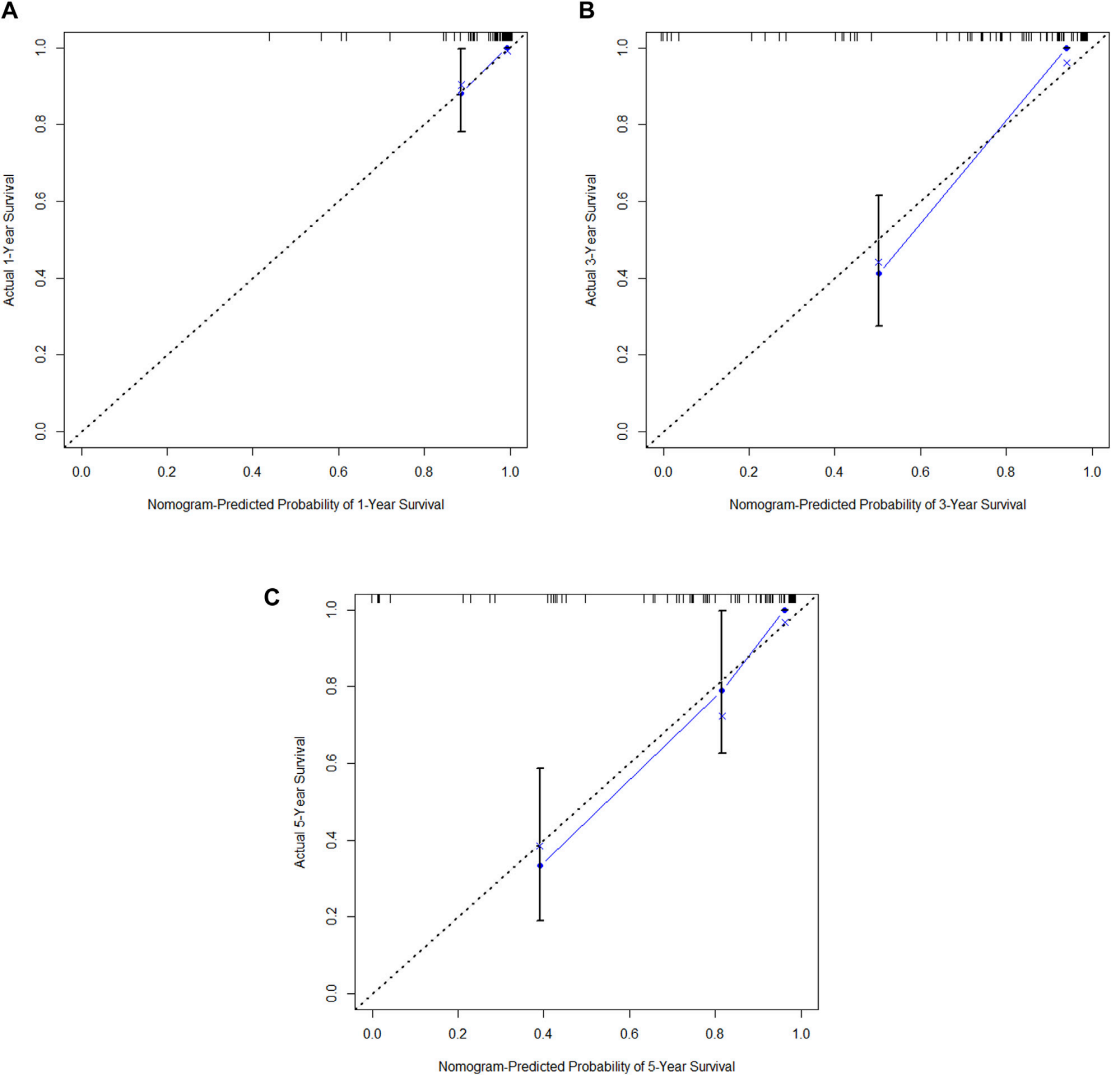
**(A)** Calibration curve of the validation set at 1 year. **(B)** Calibration curve of the validation set at 3 year. **(C)** Calibration curve of the validation set at 5 year.

In the training cohort, Kaplan-Meier estimates were used to generate DFS curves based on different demographic, clinical-pathological, and treatment factor values, which were compared using the log-rank test. The variables selected in the final multivariate Cox regression model included ypN3, high postoperative sTIL expression, receipt of postoperative radiotherapy, and effective neoadjuvant chemotherapy (see Table 3). A nomogram was then created that incorporated these four prognostic variables, which we named the nomogram for predicting disease-free survival in triple-negative breast cancer patients undergoing neoadjuvant chemotherapy (Figure 6). Each subtype of these variables was assigned a score. In simple terms, specific values for TNBC patients can be input into the nomogram to calculate their score. Based on this score, we can predict the individual’s 1-year, 3-year, and 5-year disease-free survival times. The area under the curve (AUC) of the nomograms in both the training and validation groups achieved ideal consistency, with C-indices of 0.895 and 0.865 respectively. The AUCs for 1-year, 3-year, and 5-year survival were 0.863, 0.966, and 0.971 (Figure 7) and 0.946, 0.932, and 0.949 (Figure 8), with the corresponding calibration curves for 1-year, 3-year, and 5-year respectively displayed in Figures 9A–C, 10A–C.

**TABLE 3:** Univariate and Multivariate Analysis of DFS in the training set TNBC patients.

Variables	N	Events (%)	Univariate analysis		Multivariate analysis	
			HR (95%CI)	P	HR (95%CI)	P
Age at diagnosis						
≤35 years	33 (18.97)	13 (24.53)	ref			
>35 years	141 (81.03)	40 (75.47)	0.665 (0.356–1.245)	0.202		
Menstrual status						
Premenopausal	106 (60.92)	36 (67.92)	ref			
Postmenopausal	68 (39.08)	17 (32.08)	0.701 (0.393–1.248)	0.227		
Family history						
No	153 (87.93)	49 (92.45)	ref			
Yes	21 (12.07)	4 (7.55)	0.554 (0.2–1.534)	0.256		
Surgical approach						
Radical surgery	151 (86.78)	49 (92.45)	ref			
Breast-conserving surgery	23 (13.22)	4 (7.55)	0.463 (0.167–1.284)	0.139		
ypT staging						
ypT0/Tis	58 (33.33)	16 (30.19)	ref			
ypT1	56 (32.18)	11 (20.75)	0.627 (0.291–1.35)	0.233		
ypT2	37 (21.26)	16 (30.19)	1.698 (0.849–3.396)	0.135		
ypT3	21 (12.07)	9 (16.98)	1.586 (0.701–3.588)	0.269		
ypT4	2 (1.15)	1 (1.89)	1.871 (0.248–14.124)	0.544		
ypN staging						
ypN0	103 (59.2)	11 (20.75)	ref		ref	
ypN1	40 (22.99)	18 (33.96)	4.977 (2.349–10.549)	<0.001	1.202 (0.462–3.124)	0.706
ypN2	18 (10.34)	11 (20.75)	7.304 (3.158–16.894)	<0.001	1.805 (0.630–5.174)	0.271
ypN3	13 (7.47)	13 (24.53)	35.677 (15.137–84.089)	<0.001	7.395 (2.303–23.749)	0.001
Post-histological grading						
G1+G2	113 (64.94)	31 (58.49)	ref			
G3	61 (35.06)	22 (41.51)	1.347 (0.78–2.326)	0.285		
Post-lymph-vascular invasion						
No	124 (71.26)	23 (43.4)	ref		ref	
Yes	50 (28.74)	30 (56.6)	4.382 (2.54–7.56)	<0.001	1.192 (0.597–2.382)	0.619
Post-sTIL levels						
Low	46 (26.44)	30 (56.6)	ref		ref	
Intermediate	40 (22.99)	19 (35.85)	0.511 (0.288–0.91)	0.023	0.538 (0.266–1.086)	0.084
High	88 (50.57)	4 (7.55)	0.042 (0.015–0.119)	<0.001	0.214 (0.057–0.806)	0.023
Post-Her2 levels						
0	35 (20.11)	19 (35.85)	ref		ref	
1+	95 (54.6)	23 (43.4)	0.370 (0.202–0.681)	0.001	1.461 (0.745–2.865)	0.269
2+	44 (25.29)	11 (20.75)	0.384 (0.183–0.808)	0.012	1.056 (0.461–2.421)	0.897
Post-Ki67 levels						
Ki67≤20	40 (22.99)	7 (13.21)	ref			
Ki67>20	134 (77.01)	46 (86.79)	2.197 (0.991–4.866)	0.053		
Post-CK5/6 levels						
CK5/6<1	88 (50.57)	22 (41.51)	ref			
CK5/6≥1	86 (49.43)	31 (58.49)	1.558 (0.902–2.691)	0.112		
Post-EGFR levels						
EGFR<1	86 (49.43)	22 (41.51)	ref			
EGFR≥1	88 (50.57)	31 (58.49)	1.457 (0.844–2.516)	0.177		
Post-AR levels						
<1	101 (58.05)	37 (69.81)	ref		ref	
≥1	73 (41.95)	16 (30.19)	0.553 (0.308–0.995)	0.048	0.855 (0.429–1.703)	0.655
Neoadjuvant + postoperative adjuvant chemotherapy drugs used						
Anthracyclines + taxanes	139 (79.89)	39 (73.58)	ref			
Combined with platinum	35 (20.11)	14 (26.42)	1.418 (0.769–2.615)	0.263		
Radiation therapy status						
No	30 (17.24)	21 (39.62)	ref		ref	
Yes	144 (82.76)	32 (60.38)	0.229 (0.131–0.399)	<0.001	0.3 (0.145–0.619)	0.001
NAC response						
Non-response groups	70 (40.23)	48 (90.57)	ref		ref	
Response groups	104 (59.77)	5 (9.43)	0.045 (0.018–0.113)	<0.001	0.099 (0.034–0.285)	<0.001

### 3.4 Nomogram prognostic model for overall survival (OS)

**FIGURE 11:**
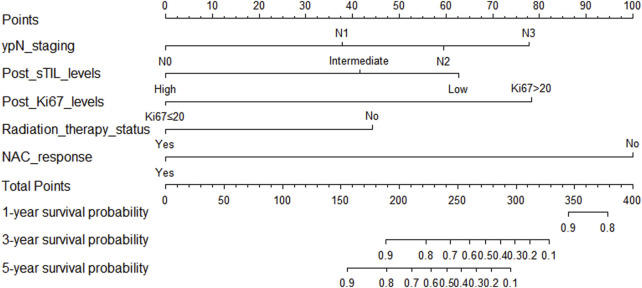
Nomogram for predicting overall survival in triple-negative breast cancer patients undergoing neoadjuvant chemotherapy.

**FIGURE 12:**
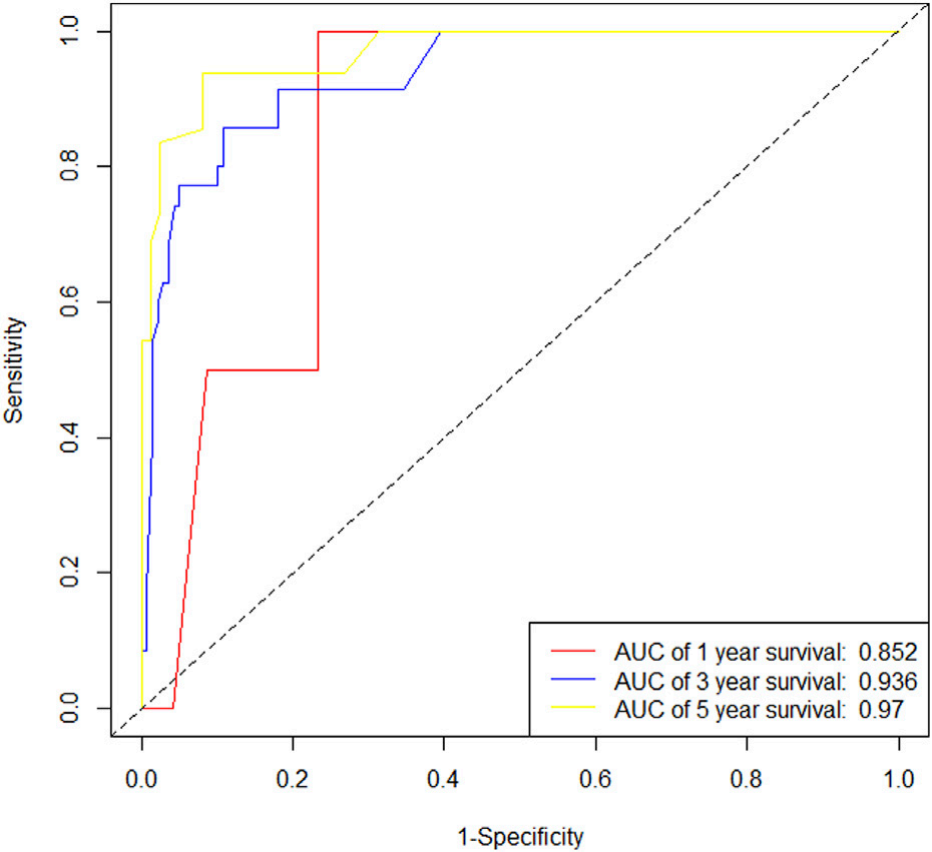
ROC curve of the training set.

**FIGURE 13:**
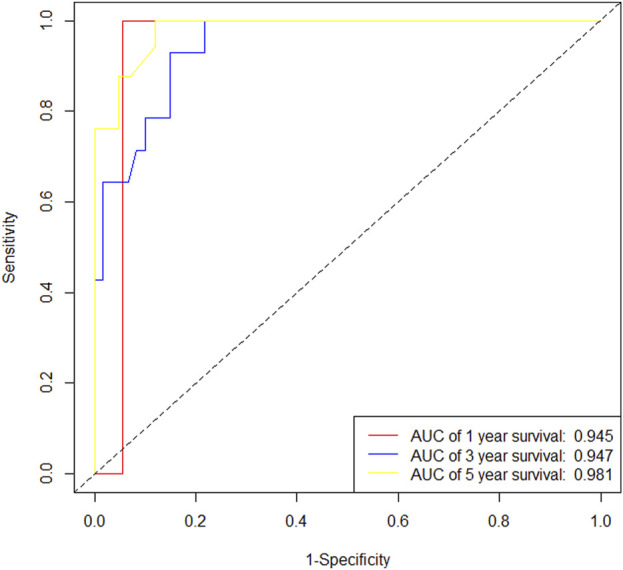
ROC curve of the validation set.

**FIGURE 14:**
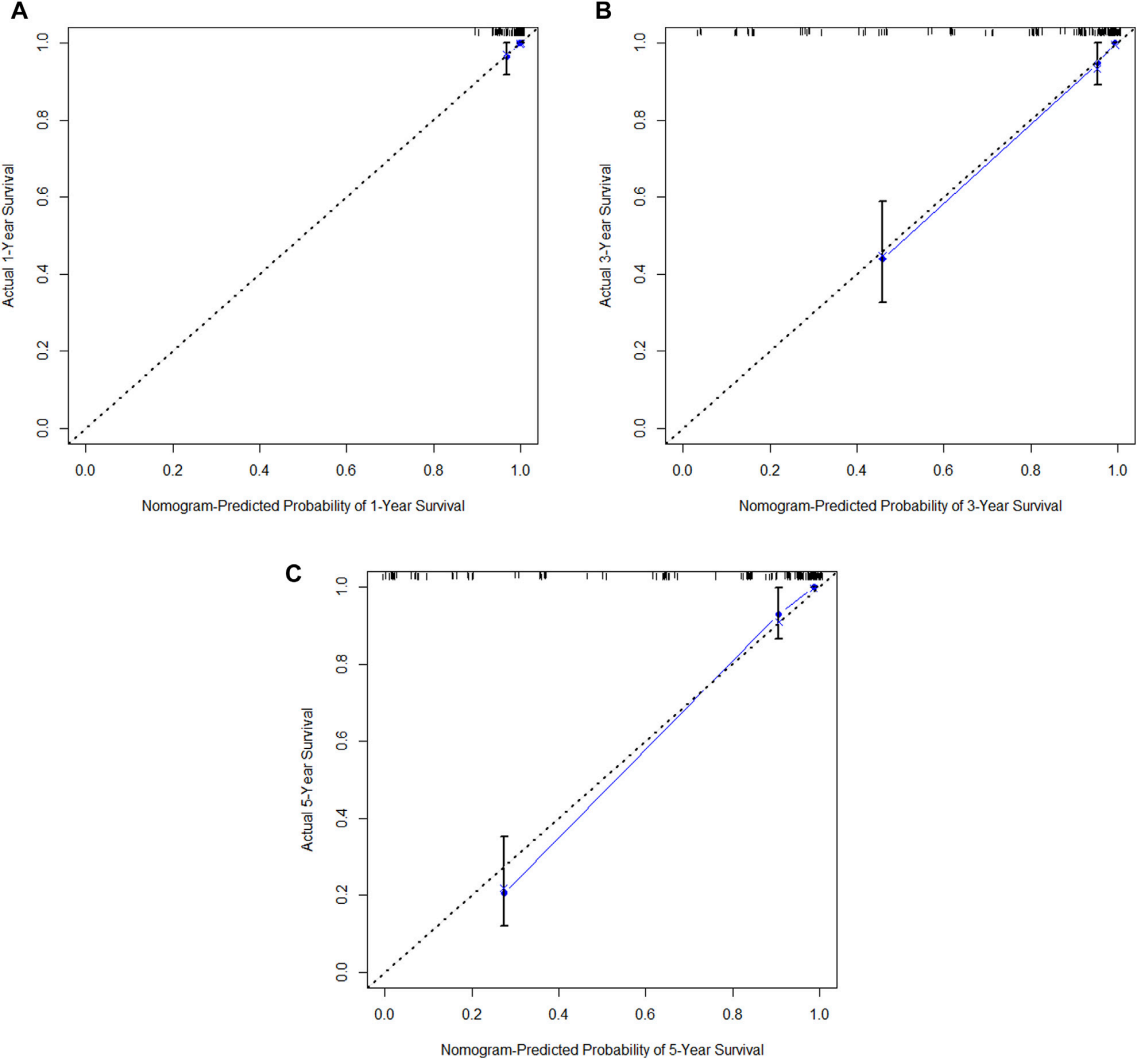
**(A)** Calibration curve of the training set at 1 year. **(B)** Calibration curve of the training set at 3 year. **(C)** Calibration curve of the training set at 5 year.

**FIGURE 15:**
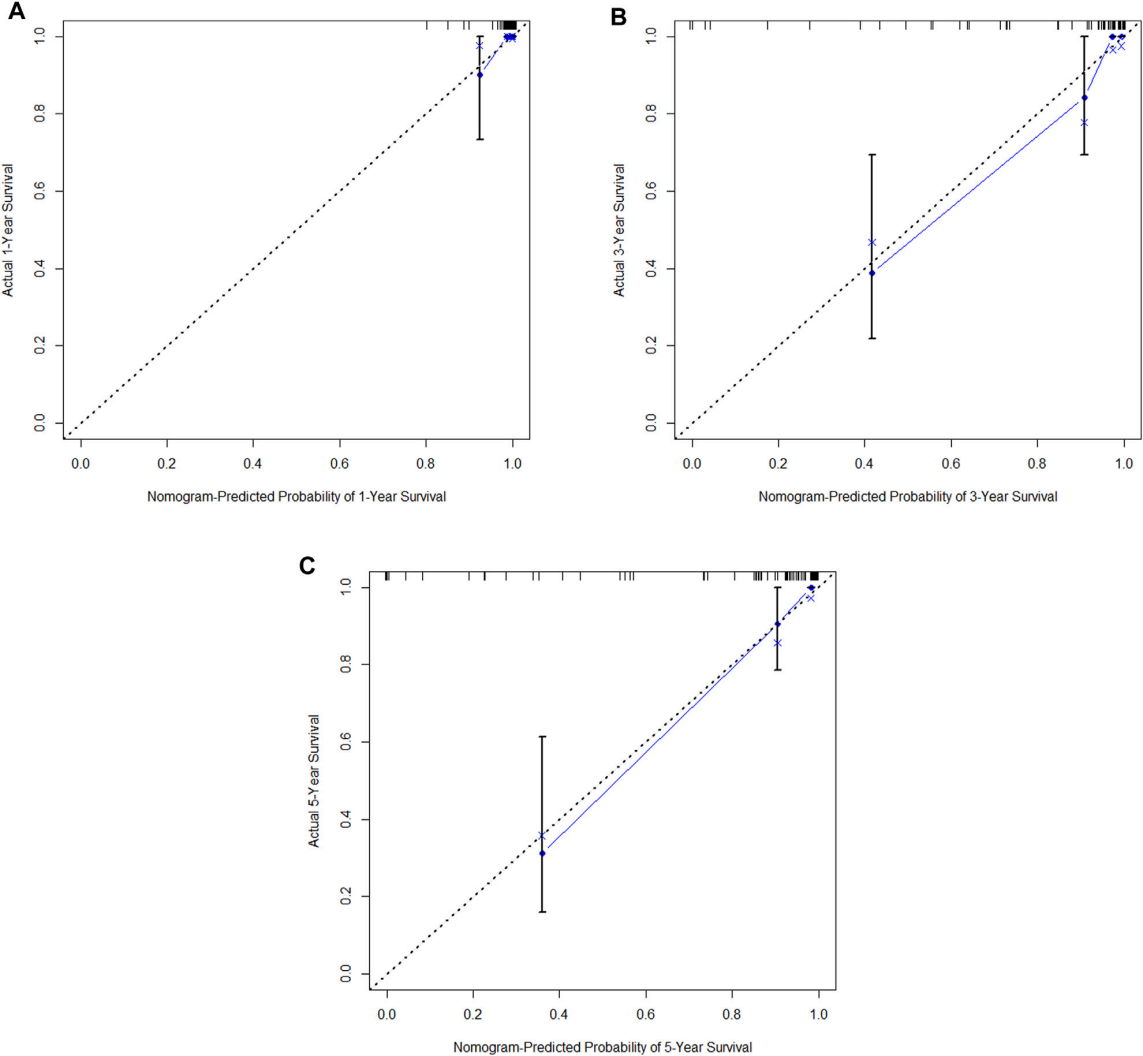
**(A)** Calibration curve of the validation set at 1 year. **(B)** Calibration curve of the validation set at 3 year. **(C)** Calibration curve of the validation set at 5 year.

In the training cohort, Kaplan-Meier estimates were used to generate OS curves based on different demographic, clinical-pathological, and treatment factor values, which were compared using the log-rank test. The variables obtained from the final multivariate Cox regression model included ypN2, ypN3, high postoperative sTIL expression, postoperative Ki67 > 20%, receipt of postoperative radiotherapy, and effective NAC (see Table 4). A nomogram incorporating these six prognostic variables was then developed, which we named the nomogram for predicting overall survival in triple-negative breast cancer patients undergoing neoadjuvant chemotherapy (Figure 11). Each subtype of these variables was also assigned a score. In simple terms, specific values for TNBC patients can be input into the nomogram to calculate their score. Based on this score, we can predict the individual’s 1-year, 3-year, and 5-year overall survival times. The AUCs of the nomograms in both the training and validation groups achieved ideal consistency, with C-indices of 0.899 and 0.860 respectively. The AUCs for 1-year, 3-year, and 5-year survival were 0.852, 0.936, and 0.970 (Figure 12) and 0.945, 0.947, and 0.981 (Figure 13), with the corresponding calibration curves for 1-year, 3-year, and 5-year respectively displayed in Figures 14A–C, 15A–C.

**TABLE 4:** Univariate and Multivariate Analysis of OS in the training set TNBC patients.

Variables	N	Events (%)	Univariate analysis		Multivariate analysis	
			HR (95%CI)	P	HR (95%CI)	P
Age at diagnosis						
≤35 years	33 (18.97)	13 (25.49)	ref			
>35 years	141 (81.03)	38 (74.51)	0.609 (0.324–1.143)	0.123		
Menstrual status						
Premenopausal	106 (60.92)	35 (68.63)	ref			
Postmenopausal	68 (39.08)	16 (31.37)	0.672 (0.372–1.215)	0.189		
Family history						
No	153 (87.93)	47 (92.16)	ref			
Yes	21 (12.07)	4 (7.84)	0.539 (0.194–1.496)	0.235		
Surgical approach						
Radical surgery	151 (86.78)	47 (92.16)	ref			
Breast-conserving surgery	23 (13.22)	4 (7.84)	0.494 (0.178–1.37)	0.175		
ypT staging						
ypT0/Tis	58 (33.33)	15 (29.41)	ref			
ypT1	56 (32.18)	11 (21.57)	0.71 (0.326–1.545)	0.388		
ypT2	37 (21.26)	15 (29.41)	1.854 (0.906–3.797)	0.091		
ypT3	21 (12.07)	9 (17.65)	1.988 (0.869–4.548)	0.104		
ypT4	2 (1.15)	1 (1.96)	3.052 (0.401–23.227)	0.281		
ypN staging						
ypN0	103 (59.2)	11 (21.57)	ref		ref	
ypN1	40 (22.99)	17 (33.33)	4.971 (2.321–10.649)	<0.001	2.147 (0.8–5.764)	0.129
ypN2	18 (10.34)	11 (21.57)	8.67 (3.735–20.124)	<0.001	3.344 (1.067–10.48)	0.038
ypN3	13 (7.47)	12 (23.53)	19.728 (8.559–45.471)	<0.001	5.255 (1.685–16.393)	0.004
Post-histological grading						
G1+G2	113 (64.94)	29 (56.86)	ref			
G3	61 (35.06)	22 (43.14)	1.493 (0.856–2.604)	0.158		
Post-lymph-vascular invasion						
No	124 (71.26)	22 (43.14)	ref		ref	
Yes	50 (28.74)	29 (56.86)	4.835 (2.759–8.474)	<0.001	1.334 (0.643–2.769)	0.439
Post-sTIL levels						
Low	46 (26.44)	29 (56.86)	ref		ref	
Intermediate	40 (22.99)	18 (35.29)	0.584 (0.324–1.053)	0.074	0.604 (0.309–1.181)	0.141
High	88 (50.57)	4 (7.84)	0.043 (0.015–0.123)	<0.001	0.245 (0.07–0.863)	0.029
Post-Her2 levels						
0	35 (20.11)	19 (37.25)	ref		ref	
1+	95 (54.6)	21 (41.18)	0.342 (0.184–0.637)	0.001	1.249 (0.634–2.461)	0.521
2+	44 (25.29)	11 (21.57)	0.4 (0.19–0.842)	0.016	1.592 (0.661–3.836)	0.3
Post-Ki67 levels						
Ki67≤20	40 (22.99)	6 (11.76)	ref		ref	
Ki67>20	134 (77.01)	45 (88.24)	2.723 (1.161–6.385)	0.021	5.598 (2.063–15.19)	0.001
Post-CK5/6 levels						
CK5/6<1	88 (50.57)	22 (43.14)	ref			
CK5/6≥1	86 (49.43)	29 (56.86)	1.477 (0.848–2.571)	0.168		
Post-EGFR levels						
EGFR<1	86 (49.43)	22 (43.14)	ref			
EGFR≥1	88 (50.57)	29 (56.86)	1.396 (0.802–2.431)	0.238		
Post-AR levels						
<1	101 (58.05)	36 (70.59)	ref			
≥1	73 (41.95)	15 (29.41)	0.59 (0.322–1.081)	0.088		
Neoadjuvant + postoperative adjuvant chemotherapy drugs used						
Anthracyclines + taxanes	139 (79.89)	37 (72.55)	ref			
Combined with platinum	35 (20.11)	14 (27.45)	1.615 (0.873–2.989)	0.127		
Radiation therapy status						
No	30 (17.24)	20 (39.22)	ref			
Yes	144 (82.76)	31 (60.78)	0.233 (0.132–0.41)	<0.001	0.341 (0.172–0.678)	0.002
NAC response						
Non-response groups	70 (40.23)	46 (90.2)	ref		ref	
Response groups	104 (59.77)	5 (9.8)	0.049 (0.019–0.124)	<0.001	0.099 (0.036–0.273)	<0.001

## 4 Discussion

It is well known that triple-negative breast cancer (TNBC) is the subtype of breast cancer with the poorest prognosis. Neoadjuvant therapy plays a significant role in locally advanced TNBC, serving as an important method for tumor downstaging. More importantly, it provides valuable information regarding drug sensitivity. For patients who do not achieve pathological complete response, close follow-up or early intervention with intensified, exploratory treatments (such as capecitabine intensification therapy, metronomic therapy, or combination with other drugs like PARP inhibitors, etc.) can further improve outcomes for this group of patients (Masuda et al., 2017; Wang et al., 2021; Tutt et al., 2021). Therefore, evaluating the efficacy of neoadjuvant chemotherapy is crucial for clinical decision-making by healthcare providers.

For aggressive breast cancer subtypes, particularly TNBC, pathological complete response (pCR) is considered an important surrogate marker for good prognosis (Conforti et al., 2021; Cortazar et al., 2014; van den Ende et al., 2023). Many studies have explored whether clinical-pathological features of tumors, such as tumor size, histological grade, and lymph node involvement, can serve as predictive biomarkers for the efficacy of neoadjuvant chemotherapy, but the results have been inconsistent. For instance, some studies found an association between smaller tumor size, lower histological grade, and pCR rates, while other articles reported no such correlations (van den Ende et al., 2023; Guestini et al., 2019; Kedzierawski et al., 2021). In our study, based on the clinical-pathological parameters we obtained, we found that high biopsy-sTILs expression, biopsy-Ki67 > 20%, and positive expression of biopsy-androgen receptor were positively correlated with the pCR rate following neoadjuvant chemotherapy. We hypothesize that this may be due to the higher proliferative activity and increased expression of infiltrating lymphocytes in tumor cells, which are more readily recognized and eliminated by the immune system, particularly the abundant anti-tumor immune cells within the infiltrating lymphocytes, such as CD4^+^ T cells and CD8^+^ T cells (Zhang et al., 2021; Sun et al., 2022). However, this requires further validation through basic research. The positive correlation between positive expression of biopsy-androgen receptor and pCR rate differs from previous related studies (Shi et al., 2023), possibly due to the small sample size in this study; future research will need to expand the sample size for further verification.

As an aggressive and highly heterogeneous subtype of breast cancer, although neoadjuvant chemotherapy has been widely applied in the treatment of triple-negative breast cancer (TNBC) in clinical practice, predicting prognosis after neoadjuvant therapy still presents challenges, especially for patients with residual disease. Currently, there is a lack of effective and clinically practical prognostic indicators. Previous studies have identified several key factors affecting the prognosis of TNBC patients after neoadjuvant chemotherapy, including the level of tumor-infiltrating lymphocytes, PD-L1 expression, tumor size, lymph node status, chemotherapy regimens, and response to treatment (Pinard et al., 2020; Fisher et al., 2024; Sarradin et al., 2021; Zhu et al., 2021; Zhu et al., 2022; Liu et al., 2024; Huang et al., 2020a; Huang et al., 2020b). In our study, based on the clinical pathological parameters obtained, we found that the factors predicting disease-free survival (DFS) in TNBC patients after neoadjuvant chemotherapy are ypN3, high postoperative sTIL expression, receipt of postoperative radiotherapy, and effective NAC, while the factors predicting overall survival (OS) are ypN2, ypN3, high postoperative sTIL expression, postoperative Ki67 > 20%, receipt of postoperative radiotherapy, and effective NAC.

Combining the results of our study, we analyzed that compared to other clinical pathological parameters, lymph node metastasis post-surgery is an important risk factor for prognosis. We believe the main reason is that lymphatic metastasis is one of the most common forms of metastasis in breast cancer patients, and once lymph node metastasis occurs, patients may face hematogenous metastasis and distant organ involvement. Therefore, its impact on prognosis often serves as a more dangerous signal, especially when the efficacy of neoadjuvant chemotherapy is poor. For tumor patients with active cellular proliferation and high expression of infiltrating lymphocytes in the stroma, this may indicate that tumor cells are more likely to be monitored and cleared by the body’s anti-tumor immunity, particularly through anti-tumor immunotherapy. Additionally, in this study, we found that patients who completed neoadjuvant chemotherapy and received postoperative radiotherapy could further improve their overall survival, aligning with findings from most current research (Gradishar et al., 2024; Abdel-Wahab et al., 1998; Huang et al., 2004; McGuire et al., 2007; Swisher et al., 2016). We believe that for most patients initially receiving neoadjuvant chemotherapy, their tumor burden is likely significant, indicating locally advanced disease. Thus, decisions regarding postoperative radiotherapy should consider the maximum stage of the disease at initial diagnosis (such as clinical stage, pathological stage, and tumor characteristics), as well as the pathological results after neoadjuvant therapy. Even if pathological complete response is achieved post-neoadjuvant therapy, postoperative radiotherapy may still enhance local control of the tumor, thereby improving patient prognosis; however, this warrants further exploration and analysis in future studies.

## 5 Limitations

Limitations of Our Study: 1) The retrospective nature of the study carries an inherent possibility of selection bias. 2) The clinical T staging of patients before neoadjuvant therapy relied on imaging assessment, lacking clear definitions for the invasive T stage, and for patients classified as cN0, there is a possibility of false negatives due to the small sample size from biopsy specimens, which may not adequately reflect the local situation. 3) The total number of patients included in the study and the treatment centers involved are relatively small, covering only the Chinese population. Whether these findings are applicable to Western populations remains to be further validated, and more external validation is needed to test the reliability and accuracy of the nomogram. Future studies should aim to expand the sample size by incorporating cross-regional, cross-national, and cross-ethnic data to better analyze the treatment efficacy and predictive biomarkers related to prognosis in triple-negative breast cancer patients receiving neoadjuvant chemotherapy.

## 6 Conclusion

Although there have been several studies on nomogram models predicting the efficacy of neoadjuvant chemotherapy and prognostic biomarkers for triple-negative breast cancer, there remains a lack of widely accepted, practical, and actionable biomarkers in clinical practice (Fisher et al., 2024; Wang et al., 2023; Zhou et al., 2024). This study establishes and validates a nomogram model based on tumor biopsy results from patients undergoing NAC, postoperative pathological findings, and relevant clinical data readily available from patients. Our aim is to assist clinicians in more easily identifying patients who will respond favorably or unfavorably to treatment. We hope that our model can contribute to the precision and individualized treatment of patients with TNBC. However, further validation with larger patient cohorts is still necessary to confirm the robustness of our findings.

## Data Availability

The original contributions presented in the study are included in the article/supplementary material, further inquiries can be directed to the corresponding authors.

## Abbreviations

TNBC, Triple-negative breast cancer; ER, Estrogen receptor; PR, Progesterone receptor; HER2, Human epidermal growth factor receptor 2; NAC, Neoadjuvant chemotherapy; pCR, Pathological complete response; AR, Androgen receptor; DFS, Disease-free survival; OS, Overall survival; EFS, Event-free survival; sTIL, Stromal tumor-infiltrating lymphocytes; IHC , Immunohistochemical; ASCO:American Society of Clinical Oncology; CAP, College of American Pathologists; FISH, Fluorescence in situ hybridization; CK5/6, Cytokeratin 5/6; EGFR, Epidermal growth factor receptor; RECIST, Response evaluation criteria in solid tumors; CR, Complete response; PR, Partial response; PD, Progressive disease; SD, Stable disease; AUC, The area under the curve; PARP inhibitor, Poly(ADP-ribose) polymerase Inhibitor.
